# Problem Space Matters: Evaluation of a German Enrichment Program for Gifted Children

**DOI:** 10.3389/fpsyg.2018.00569

**Published:** 2018-04-23

**Authors:** Marisete M. Welter, Saskia Jaarsveld, Thomas Lachmann

**Affiliations:** ^1^Cognitive and Developmental Psychology, Center for Cognitive Science, University of Kaiserslautern, Kaiserslautern, Germany; ^2^Master Program for Psychology, Potiguar University, Natal, Brazil; ^3^Experimental Psychology Unit, University of Leuven, Leuven, Belgium

**Keywords:** cognitive development, giftedness, reasoning, childhood, gifted education, problem space

## Abstract

We studied the development of cognitive abilities related to intelligence and creativity (*N* = 48, 6–10 years old), using a longitudinal design (over one school year), in order to evaluate an Enrichment Program for gifted primary school children initiated by the government of the German federal state of Rhineland-Palatinate (*Entdeckertag Rheinland Pfalz*, Germany; ET; Day of Discoverers). A group of German primary school children (*N* = 24), identified earlier as intellectually gifted and selected to join the ET program was compared to a gender-, class- and IQ- matched group of control children that did not participate in this program. All participants performed the Standard Progressive Matrices (SPM) test, which measures intelligence in well-defined problem space; the Creative Reasoning Task (CRT), which measures intelligence in ill-defined problem space; and the test of creative thinking-drawing production (TCT-DP), which measures creativity, also in ill-defined problem space. Results revealed that problem space matters: the ET program is effective only for the improvement of intelligence operating in well-defined problem space. An effect was found for intelligence as measured by SPM only, but neither for intelligence operating in ill-defined problem space (CRT) nor for creativity (TCT-DP). This suggests that, depending on the type of problem spaces presented, different cognitive abilities are elicited in the same child. Therefore, enrichment programs for gifted, but also for children attending traditional schools, should provide opportunities to develop cognitive abilities related to intelligence, operating in both well- and ill-defined problem spaces, and to creativity in a parallel, using an interactive approach.

## Introduction

Early concepts of giftedness were solely based on the construct of intelligence (e.g., Terman, 1916). Even today, in practice, a single IQ-threshold of 130 is commonly used for the diagnosis of giftedness (Robinson, 2005; Sternberg et al., 2011). Assuming that, across individuals in a population, intelligence is normally distributed according to this criterion, i.e., two standard deviations above the average of IQ = 100, between 2 and 3% of the population should be considered intellectually gifted.

Giftedness later became a more complex concept and is now considered a multifaceted construct. Rather than with a single measure of intelligence (Reis and Renzulli, 2011), other abilities should be considered as well (e.g., Brown et al., 2005; Reis and Renzulli, 2011; Sternberg et al., 2011). Many theories embrace creativity as an essential component of giftedness (e.g., Runco, 1993); “creativity moves from being a background player to occupying a key role within the context of giftedness and gifted education” (Kaufman et al., 2012, p. 60). For example, two of the most prominent theories of giftedness include the construct of creativity: Renzulli’s Three-Ring Conception (Renzulli, 1978, 2005) and Sternberg’s WICS Model of Giftedness (Sternberg, 2005a,b, 2010).

Some authors argued that there may be different categories or types of giftedness (Renzulli, 1982, 2005; Milgram, 1990; Sternberg, 1990). Sternberg (1990), for instance, suggests that it is necessary to go beyond the view of giftedness as a composite of components, to the view where there are types of giftedness with multiple components each. Renzulli (1982) suggests that there are two types of giftedness: *the schoolhouse giftedness* and *the creative-productive giftedness*, whereas Milgram (1990) suggests that there are four different categories of giftedness: *general intellectual ability or overall general intelligence*, *specific intellectual ability*, *general original/creative thinking*, and *specific creative talent*.

Although there is lack of agreement concerning the concept of giftedness, there is broad consensus among researchers about the importance of giftedness education (Robinson, 2005). Gifted students require challenging educational experiences with other gifted students in order to learn and develop at their own level of aptitude (Mönks and Heller, 1994; Feldhusen, 2005; Robinson, 2005). Reviewing literature on the topic, Reis and Renzulli (2011) reported two main aspects regarding the necessity for developing special programs for gifted children: (1) their needs have not been met in traditional school curricula; (2) they benefit from programs that group them together with the educational purpose to meet their needs.

Interventions with gifted students can be made by offering programs of acceleration and enrichment within homogeneous or heterogeneous grouping (Mönks and Heller, 1994). The acceleration program usually offers learning activities that take into consideration the child’s abilities and speed of processing level, which probably will differ from that of his or her classmates; whereas the enrichment program commonly comprises activities that are, or at least should be, challenging and that go wider and deeper on the topics presented. The activities should cover subjects that typically are not addressed in traditional school curriculum (Mönks and Heller, 1994). According to Reis and Renzulli (2011), a combination of both acceleration and enrichment programs is the best option. Nevertheless, along with teaching methods and curriculum, the evaluation of the program needs to be included among the central issues for improving the quality of education offered to gifted students (Gallagher, 1988). According to Hunsaker and Callahan (1993), the evaluation of a giftedness program should offer meaningful feedback to improve the program’s performance.

While not all children have the capacity to delve wider and deeper into the topics, improving the quality of education in terms of challenging activities and consideration for individual abilities is essential to all learners and should not be restricted to gifted children. The present study, however, aims to evaluate an enrichment program designed for children identified as gifted, the *Entdeckertag Rheinland-Pfalz* (ET; Day of Discoverers). The ET is a pilot program implemented by the Ministry for Education, Science, Youth and Culture of the German federal state of Rhineland-Palatinate (in the southwest of Germany) in an attempt to recognize and support gifted children in primary school (see section “Materials and Methods”).

Since intelligence and creativity are considered as important components of giftedness, we applied a standardized test of general intelligence and a standardized test of creativity for the evaluation of the enrichment program, using a longitudinal design. The intelligence test measures cognitive processes operating in a well-defined problem space while the creativity test measures cognitive processes operating in an ill-defined problem space. Problem space is an abstract representation of the encountered problem in the mind of the problem solver, containing all possible and/or logical steps to be taken in order to find a final solution (Newell and Simon, 1972). Whereas in well-defined problem space there is only one correct solution, in an ill-defined problem space there are numerous and more idiosyncratic solutions, which can, depending on a certain criterion, be more or less good. Apart from measuring cognition in different problem spaces, the two tests use different knowledge domains: in the intelligence, test cognition operates in the domain of relations between geometrical components, in the creativity test, cognition operates in an idiosyncratic domain. Therefore, we additionally applied the Creative Reasoning Task (CRT; Jaarsveld et al., 2010, 2012). The CRT measures intelligence in an ill-defined problem space while operating in the same knowledge domain then the general intelligence test used in the present study.

Studies with the CRT showed, firstly, that scores from a task measuring intelligence in a well-defined problem space (Standard Progressive Matrices; SPM, Raven, 1998) do not correlate with scores from a task measuring intelligence in an ill-defined problem space (CRT), although both tasks use an identical knowledge domain: relations among geometrical components in a small matrix. In this first version of the CRT, scores are based on the frequency of relations applied in the matrix. Results showed that 4–12-year-old children applied these relations with different frequencies depending on the task (Jaarsveld et al., 2010).

Secondly, Jaarsveld et al. (2012) showed that in the CRT both convergent and divergent thinking are applied and can be assessed independently. This second version of the CRT contained a score for convergent and one for divergent thinking. The convergent sub-score valuates not only the number of relations but also their complexity and whether a relation was applied over all rows and columns of the matrix. This method produces a larger range of score values and therefore provides more differentiated information of reasoning abilities. Using this new scoring method it was shown that from Grade 1 to 4 the CRT sub-score for convergent production correlated with the SPM while the CRT sub-score for divergent production correlated with the Test for Creative Thinking–Drawing Production (TCT–DP, *Test zum Schöpferisches Denken–Zeichnerisch*, Urban and Jellen, 1995). This suggests that intelligence measured for ill-defined problem space cooperates with creative thinking. This cooperation is also evident in EEG data from the CRT process in terms of an intertwining of convergent and divergent production (Jaarsveld et al., 2015). These results show that intelligence in ill-defined problem space is distinct from intelligence in well-defined space, even when knowledge domain is controlled.

Finally, Welter et al. (2017) found that across primary school cognitive processes in well-defined problem space develop differently from those in ill-defined space. They showed that traditional intelligence test scores (well-defined problem space) increased linearly with grade level, whereas scores from a creativity test (TCT-DP) and those from the CRT sub-score for convergent production (both ill-defined problem space) developed in the same irregular pattern.

From these studies, we can conclude that systematic comparisons between intelligence operating in well- versus ill-defined problem space are more meaningful when cognition in both problem spaces operates in an identical knowledge domain. This, however, is not the case in the majority of studies comparing measures from traditional intelligence and creativity tests (see Jaarsveld and Lachmann, 2017, for an overview).

In sum, the main purpose of the present study is to evaluate the effectiveness of the ET program in improving intelligence and creative abilities of primary school children over the stretch of one school year. We applied three tests, one test measuring intelligence in a well-defined problem space and one test measuring intelligence in an ill-defined problem space, both using the same knowledge domain, and the third one, measuring creativity in an ill-defined problem space. An increase in performance on all three tests would indicate that the ET program adjusted its teaching method and curriculum, promoting cognitive abilities that allow an individual to effectively navigate and operate in both well- and ill-defined problem spaces.

## Materials and Methods

### Enrichment Program

#### General Proposal and Guidelines

The *Entdeckertag Rheinland-Pfalz (ET;* Day of Discoverers in Rhineland Palatinate) is an enrichment program for gifted children. It was implemented in 2004 by the Ministry for Education, Science, Youth and Culture of the federal state of Rhineland-Palatinate, Germany. The ET takes place on one fixed day of the week, from 8 am to 4 pm. On this day participating children (ET-children), instead of their normal class, they attend one of the ET classes. The program is conducted in select primary schools across the state, which agreed to offer the training to the ET-children, including those from different schools of the region, in addition to their traditional education scheme. This means that in order to participate the majority of the ET-children need to visit a different school once per week. The ET-children are supposed to catch up on what was taught that day in their normal class. The ET classes are of mixed grade levels, divided only roughly by age into “younger” (5–7/8 years) and “older” (7/8–10 years). A statistical analyses for 2010/2011 of the federal ministry (Ministry for Education, Science, Youth and Culture of the State Rhineland-Palatinate, 2011) showed that over 6 years 424 children (276 males and 148 females) from 206 primary schools participated in the program at 13 ET schools.

The main objective of the ET program, according to the ministry (Ministry for Education, Science, Youth and Culture of the State Rhineland-Palatinate, 2009), is the early intervention for children with exceptional cognitive abilities to give support and pose challenges in the areas of language and science. According to the ministry, ET-children should experience suitable learning environments that support their cognitive abilities, promote their personality development and strengthens teamwork and social skills.

The ministry provides guidelines for the selection processes and general rules and aims as a kind of curriculum for ET classes (Ministry for Education, Science, Youth and Culture of the State Rhineland-Palatinate, 2009). These include a general agenda about the structure and the course of a day that the participating schools should follow (see **Table 1**). However, schools are free to develop their own proposal within the program’s scope. For example, the ET curriculum requires that children learn an additional foreign language that is not part of the traditional school curriculum, such as Russian or Japanese. What language this will be is, however, the choice of the ET school. Each ET school forms a team of experts that is responsible for the local implementation and organization of the ET program and the diagnostic selection process. This ET team consists of teachers that were previously trained to meet the purpose of the program and to recognize and foster an active, creative, and inquisitive thinking attitude among the gifted children.

**Table 1:** Daily structure of the *Entdeckertag* Program.

Time		
From	To	Daily Structure^∗^
8:00	09:30	Work on Topic 1 (mathematics, natural sciences or German language)
09:30	10:30	Work on Topic 2 (language learning or task packages)
10:30	12:00	Research-based learning to self-selected projects
12:00	13:00	Sports, games, planned leisure activities, reading or computer work
13:00	13:30	Lunch and free time
13:30	13:45	Outdoors activities
13:45	16:00	Afternoon projects possibly with extracurricular experts

*^∗^Time interval can be interpreted as a flexible framework for the Entdeckertag. This also applies to the intermediate brake times that are not listed separately. Adapted from “Erkennen und Fördern hochbegabter Kinder in der Grundschule: Entdeckertag – Modellprojekt des Landes Rheinland-Pfalz” by Ministry for Education, Science, Youth and Culture of the State Rhineland-Palatinate (2009), www.grundschule.bildung-rp.de.*

In Germany, every child receives free education, which is of high quality, but mainly uses a traditional teacher-directed lesson format. In contrast to this traditional schooling, the ET program aims to offer a variety of more *self-directed learning* opportunities. The focus is on individual “research projects”; each child chooses one topic within a given scope. The child should be able to structure the gathered information and present it to classmates and parents as a poster, including pictures and text, or as a power point presentation. During the research phase, children should have the opportunity to discuss issues about their topic with other children in a plenary meeting in order to get comments and suggestions (Baudson, 2009). In addition to the project work, children can work on brainteasers and applied science problems. Moreover, sports and arts are included in the curriculum in order to promote an integrated educational approach (Emrich et al., 2007).

An important element of the ET curriculum are the so called “work packages.” These consist of tasks and activities that the children bring with them to their normal classes to work on during the rest of the week. These include reading and writing tasks, puzzles and arithmetic problems. Gifted children might feel bored during their normal classes because they usually already have a rich knowledge on many topics. Thus, the work packages are helpful in providing continuous challenges to these children. Moreover, these packages help to establish a bridge between both learning environments normal class and ET class, as the child takes the work packages back to traditional school and works on them after finishing regular activities. The teacher can also use this supplemental material as a challenge for children who are not in the ET program. Thus, the work packages help teachers to offer challenging learning activities to all children and therefore enriches future teaching in normal classes independent from the ET program (Emrich et al., 2007).

The selection process is carried out by the ET team of each school. The steps are as follows (Baudson, 2009; Ministry for Education, Science, Youth and Culture of the State Rhineland-Palatinate, 2009): (1) The parents, and/or the relevant teacher, who assume a child to be gifted, should contact an ET-school and provide necessary information to the school in order to start the selection process. Next, parents would be asked to complete a parental questionnaire and the teacher would be asked to complete a screening questionnaire about the child under consideration. These questionnaires were constructed especially for the ET selection process (Ministry for Education, Science, Youth and Culture of the State Rhineland-Palatinate, 2009). Both questionnaires include a checklist and a set of open questions about behavioral and motivational aspects of the child. The parental questionnaire also includes questions about early cognitive development and asks for proof of extraordinary cognitive performances (e.g., school certifications, rewards and other certificates of performance). It is not explained in the guidelines (Ministry for Education, Science, Youth and Culture of the State Rhineland-Palatinate, 2009) why parents have the opportunity to start the selection process and contribute to the screening, and to what extent educators can trust parents to report on their children’s cognitive development and performance. (2) Based on these screenings, the ET team decides whether the child might be eligible for the program and if so, sets an appointment for an interview with the parent(s) and the child. The ET team performs a structured interview with the parents and the child during which parents are asked about the child’s interests and social and motivational aspects. During this interview, the child is asked to perform some challenging tasks in the areas of language, mathematics and logical thinking. These tasks require spatial thinking, text comprehension, memory, reasoning, conceptual thinking and number processing skills. Within the interview the number and type of tasks given is attuned to the performance of the child. (3) In a separate meeting, the ET team makes a decision for each child.

#### The Present ET Sample

The present study was conducted in a school which in 2009 began participating in the ET program for gifted children in the city of Kaiserslautern (a major city in the German federal state of Rhineland-Palatinate) and neighboring communities. At this particular school, the ET took place Wednesdays, from 8 am to 4 pm. The school provided lunch, drinks, and fruits and vegetables as snacks for all participating children throughout the day. The children were divided into two groups: Group 1, first and second graders, and Group 2, third and fourth graders, i.e., in this ET school, rather than age, the grade level was used as the criterion.

The local implementation and organization of the ET program and the daily schedule followed the guidelines and general rules given by the ministry (for daily schedule see **Table 2**). Every Wednesday the ET class started with children from both groups together with a discussion about the previous week’s work packages, including their feedback on where and with whom they worked on it, and how they liked it. After, there was an open debate on a curriculum-related topic chosen by the teachers or the children.

**Table 2:** Hosted School daily schedule.

Time		Daily Schedule
From	To	
8:00 a.m.	10:00 a.m.	Debate on a topic selected by teachers and/or students and work packages^∗^
10:00 a.m.	11:00 a.m.	Russian lessons
11:00 a.m.	12:00 a.m.	“Own topic”^∗∗^
12:00 a.m.	13:00 p.m.	Reading and playing
13:00 p.m.	14:00 p.m.	Lunch and exercise (gymnastics)
14:00 p.m.	16:00 p.m.	Experiments

*^∗^Tasks that include reading and writing activities and puzzling and arithmetic problems that ET-children have to work on over the week in their normal classes. ^∗∗^Each child chooses a topic of interest to research and present to the class. They use internet to perform their research. The presentation is made in poster (Group 1) or power point (Group 2). Each child sets the time limit for their research*.

Thereafter, children were divided into the two groups. In one group, children received lessons in Russian as a foreign language (not offered in traditional schools), while the other worked on the “research projects,” and then vice-versa. The Russian lessons were held by a native speaker in the classic teacher-directed lesson format, with a focus on grammar and translation. In the research projects, using the internet, each child investigated a self-chosen topic of interest, and reported results. Next, there was reading and playing time, in which all children mingled again. At this moment, children had the opportunity to immerse themselves in a book of their choice, or they could play with brain- and strategy games, such as Rush Hour, Chocolate fix, Blokus, or others. After lunch and some sports activities, there was time for scientific experimentation. In this time, with all children present, some experiments were performed with the aim to raise their curiosity and interest in science.

Aside from the weekly activities, there were some excursions such as a hiking/climbing day, a visit to the Technical Museum in Speyer, and to the TECHNOSEUM (State Museum of Technology and Work) in Manheim, both neighboring cities in Germany.

### Participants

From the hosting primary school, a sample of 190 children from Grade 1 to Grade 4, between the ages of 6 and 10 years old, was tested. The ethical, formal, and legal standards of the study were approved by the *Aufsichts- und Dienstleistungsdirektion Trier* (*ADD*, a federal state institution responsible for approving studies conducted in public school). The study was conducted in accordance with the recommendations of The German Society of Psychology after receiving written informed consent from the parents in accordance with the Declaration of Helsinki. Children performed the paper-and-pencil versions of the SPM (Raven, 1998), the CRT (Jaarsveld et al., 2010, 2012), and the TCT-DP (Urban and Jellen, 1995; Form A and B). For all participants, IQ was obtained using the German norms provided for the SPM (1998; 6–18 years old). Participants were divided according to two general cohorts: Intervention group (IG) and control group (CG).

The IG comprised children (*N* = 24, *M*_age_ = 8.04, *M*_IQ_ = 133.25, 18 male) who participated in the *Entdeckertag (ET)* program. The large control group was composed of children that attended the normal classes in the same primary school where the ET program took place (*N* = 166). From this pool, participants were selected according their gender, grade, IQ, creative reasoning, and creativity scores (on the basis of SPM, CRT, and TCT-DP scores from the first test session, see below) in order to build a matched control group (CG, *N* = 24, *M*_age_ = 8.00, *M*_IQ_ = 133, 18 male). This means, for each IG child, a control child attending a traditional school class at the same ET school, of the same gender, grade, and close to identical test scores was chosen. Thus, there were 48 participants in total.

### Material

#### Standard Progressive Matrices (SPM)

The SPM (Raven, 1998) is a non-verbal intelligence test which measure intelligence the traditional way, i.e., operating in well-defined problem space. The test contains 60 items grouped in five sets; each item comprising an incomplete figure-pattern presented in the form of a 1 × 1, 2 × 2, or 3 × 3 matrix. Participants are asked to complete the pattern by finding the one correct figure from six or eight possible solution options given below the matrix. The maximum score is 60 points, since each item is fixed as pass or fail. The items are, at first, easy and simple but become increasingly more difficult within and across sets, requiring higher levels of cognitive abilities to encode and analyze information (Raven, 2009). The individual test processing time and the increasing complexity of the SPM items are functional in assessing the extent of clear thinking (Heller et al., 1998).

The SPM was intended to capture the different levels of cognitive ability associated with intelligent performance in as many age groups as possible, regardless of education, nationality, or health condition (Heller et al., 1998). Raven intended to develop a test that would be theoretically relevant, easy to administer, clear to interpret, and could be administered to individuals of different ages and socio-economic backgrounds (Raven, 2009). Normally, the SPM is used from 6 years onward and all candidates have the same set of tasks in the same order (Heller et al., 1998).

#### Creative Reasoning Task (CRT)

The CRT is a diagnostic device which measures intelligence and creativity operating intertwined in an ill-defined problem space (in contrast to intelligence tests which measure intelligence the traditional way; operating in well-defined problem space, as SPM; Jaarsveld et al., 2010, 2012). The CRT involves generating components and relations which connect these components, and thus yields a cognitive thinking process in which both intelligent and creative abilities intertwine (Jaarsveld et al., 2015).

In the CRT participants have to conceive a small matrix similar to those found in the SPM. According to the instruction, the matrix must be solvable and as inventive and difficult as possible. For the present study three test forms were applied, each corresponded to one of the three possible types of matrix formats contained in the SPM: 1 × 1, 2 × 2, and 3 × 3. The figure that completes the matrix should be drawn within the matrix in the outlined square in the lower right corner. Children were free in their choice of test form. The CRT contains two sub-scores: one for intelligence in an ill-defined problem space, i.e., CRT Relations (CRT-R), and one for creativity, i.e., CRT Components & Specifications (CRT-C). Due to research question and design, the latter was not used in the present study.

CRT-R sub-score represents the logic and coherence in a pattern of components for a matrix that was created and it is evaluated by means of defined relations that can deliver up to 128 raw points. These relations are: Matrix 1 × 1 (*Idiosyncratic and Semantic Coherence, Jigsaw, and Pattern Completion*); String (*Iteration of one component, and Iteration of two or >2 components*); Matrix 2 × 2 (*Symmetry, Change, Increase, and Succession*); and Matrix 3 × 3 (*Change, Increase, Succession, Combination, Indication of Mathematical Operation, and Two Values* (see Jaarsveld et al., 2012 for detailed information). However, as the CRT deals with ill-defined space problems, it is therefore impossible to fix a set of evaluation criteria for all possible solutions.

#### Test for Creative Thinking – Drawing Production (TCT–DP)

The TCT**–**DP (Urban and Jellen, 1995) is a test for the measurement of an individual’s creative thinking potential. It can be used to identify very high creative potential as well as to recognize individuals with underdeveloped creative abilities, who may be in need of stimulation and support.

The test contains two answering forms (Form A, and Form B) both providing six figure fragments in a square frame inspiring further drawing. Based on these fragments, the respondent is requested to complete the drawing in a free and open way. Instruction emphasized that one can do nothing wrong. The fragments are: semi-circle, dot, large right angle, curved line, broken line, and a small open square outside the frame.

The drawing is evaluated and scored by means of 14 criteria that can deliver up to six raw points each, except for the four criteria of unconventionality which are valued at a maximum of three points (maximum score = 72). The criteria are: *continuations; completions; new elements; connections made with a line; connections made to produce a theme; boundary breaking that is fragment dependent; boundary breaking that is fragment independent; perspective; humor and affectivity; unconventionality A; unconventionality B; unconventionality C; unconventionality D*, and*; speed*. The test is applicable in single or group testing with individuals aged between 5 and 95 years.

### Procedure

The first test session (T1) took place shortly after the beginning of the school year and the second test (T2) session 36 weeks later, just before the end of the school year. Children were tested in groups within the time frame they would have had their normal classes. They were informed that they could end their participation at any time during the session without the need of reporting any reason. All tests were performed within one session. Children were asked to work quietly and alone. In order to facilitate this, they were seated sufficiently far away from each other. Two researchers conducted the session without the teacher being present. Children were asked firstly to perform the SPM (45 min), then to generate a SPM-style item in the CRT (20 min), and finally to complete the TCT-DP (15 min; Form A first session, Form B second session). The appropriate instruction was given to the whole group before each test. Those children who finished a test before the given time frame were allowed to read books that lay ready to this purpose.

### Data Analyses

Repeated measures ANOVAs controlled for age and *post hoc t*-tests were carried out. When more than one *t*-test was conducted for each dependent variable, multiple testing corrections were used; the *p*-value was then adjusted by the Bonferroni correction. Assumptions for the performed analyses were examined: Shapiro–Wilk test was applied to verify the normality of the sample distribution and the Levene’s test to confirm equality of variances between tests. When an assumption was violated, non-parametric statistic tests were additionally performed (namely, Mann–Whitney and Wilcoxon signed-rank tests).

## Results

Means and standard deviations of the SPM, CRT-R, and TCT-DP raw scores of the groups in the two test sessions are presented in **Table 3**.

**Table 3:** Means and (standard deviations) of raw scores of SPM, CRT-R and TCT-DP of IG and CG in T1 and T2.

Test	IG (*N* = 24)		CG (*N* = 24)	
	
	T1	T2	T1	T2
SPM	46.58 (4.93)	49.50 (4.01)	45.46 (3.35)	45.88 (4.69)
				
CRT-R	24.46 (24.14)	30.71 (23.27)	20.17 (19.38)	27.13 (19.57)
				
TCT-DP	16.63 (5.33)	15.67 (7.32)	14.58 (7.38)	14.96 (5.16)

*IG group was formed by children that were participating in the Entdeckertag program, whereas CG was the control group. SPM, Standard Progressive Matrices; CRT-R, Creative Reasoning Task – Relations sub-score; TCT-DP, Test for Creative Thinking – Drawing Production.*

**FIGURE 1:**
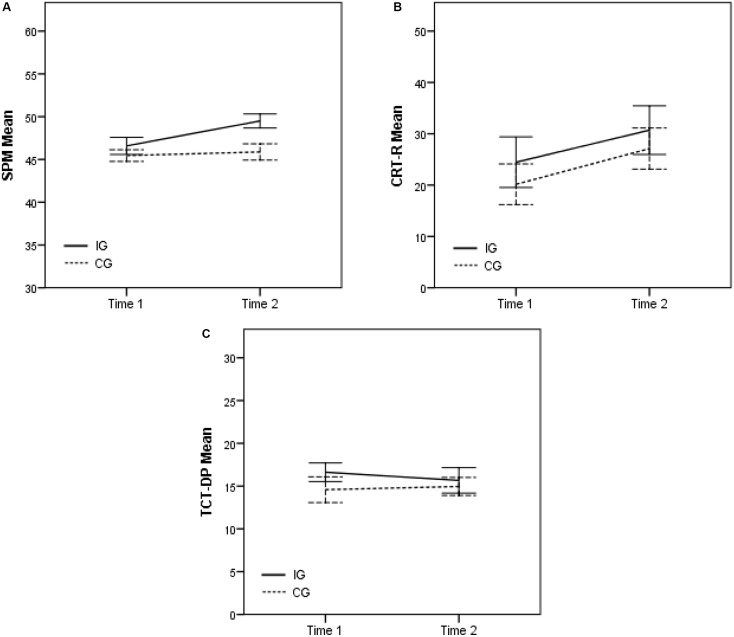
Means of the raw scores of the SPM **(A)**, CRT-R **(B)**, and TCT-DP **(C)** of IG and CG in T1 and T2.

Regarding SPM, ANOVA showed significant main effect of Group, *F*(1,45) = 6.074, *p* = 0.018, $\eta_{p}^{2}$ = 0.674 and Age, *F*(1,45) = 12.458, *p* = 0.001, $\eta_{p}^{2}$ = 0.932. Additionally, there was a significant interaction between Time and Group, *F*(1,45) = 4.227, *p* = 0.046, $\eta_{p}^{2}$ = 0.521 (**Figure 1A**). A related *t*-test (α = 0.025) revealed that the IG group had a statistically significant improvement from T1 to T2 *t*(23) = -3.436, *p* = 0.002; while there was no such effect in the CG [*t*(23) = -0.472, *p* = 0.641]. Furthermore, an independent *t*-test (α = 0.025) showed that the IG participants had higher SPM scores than the CG in T2 *t*(46) = 2.877, *p* = 0.006, whereas there is no such difference evident for T1, for which the samples were matched [*t*(46) = -0.924, *p* = 0.360).

For CRT-R, ANOVA did not show any significant effect, indicating no difference in scores between groups and time (**Figure 1B**). Because the Shapiro–Wilk test demonstrated that the normality of the data distribution cannot be assumed, non-parametric tests were performed additionally. In accordance with ANOVA: (a) The Mann–Whitney test revealed that the IG participants scores in T1 and T2 did not differ significantly from CG participants scores, *U* = 258, *z* = -0.621, *p* = 0.535 and *U* = 267, *z* = -0.434, *p* = 0.664, respectively; and (b) the Wilcoxon signed-rank test showed that there was no significant difference between the participants scores from T1 to T2, neither for IG nor for CG, *T* = 133, *p* = 0.543 and *T* = 114.25, *p* = 0.072, respectively.

Regarding TCT-DP, ANOVA did not show any significant main effect or any interaction (**Figure 1C**).

## Discussion

In the present study, we evaluated the effectiveness of an enrichment program for gifted children, the *Entdeckertag Rheinland-Pfalz (ET)*, by comparing performance of primary school children on intelligence and creativity tests over the stretch of one school year. Performance of children who participated in the ET program was compared against the performance of children who received only normal teaching. These groups were matched on gender, grade and pre-test performance (T1). Children were tested with a standardized intelligence test operating in well-defined problem space (SPM), a standardized creativity test operating in ill-defined problem space (TCT-DP), and an intelligence test that measures intelligence operating in ill-defined problem space (CRT-R).

Results showed that the ET enrichment program has a positive effect on intelligence operating in well-defined problem space, as measured by SPM. ET-children showed an increase in SPM intelligence scores while control children did not. Hence, it seems that, when referring to the traditional concept of intelligence, i.e., intelligence operating in well-defined problem space, the ET program had the appropriate teaching and curriculum to enhance children’s performance.

However, this enhancement occurred neither for creativity (TCT-DP) nor for intelligence operating in ill-defined problem space (CRT-R). Results from both of these tests measuring cognitive processes operating in ill-defined problem space showed an identical trend. This is in line with previous findings by Welter et al. (2017), showing that problem space matters. According to which of the two types of problem spaces presented, different cognitive abilities would be elicited from the same child.

According to these authors, in tasks with a well-defined problem space, different abilities are addressed than in tasks with an ill-defined space; intelligence operating in ill-defined space compares better to creativity that also unfolds in ill-defined space than to traditional intelligence. Hence, the present pattern of results together with the findings from Welter et al. (2017) indicate that problem space is an important issue when interpreting test results. The issue of different problem spaces is the most robust explanation for differences between the results of the two intelligence tests found in the present study, SPM and CRT-R, because both tests use the same knowledge domain.

From this we may infer that the enrichment program was, on the one hand, capable of supporting children in developing cognitive abilities necessary to operate in well-defined problem spaces, but, on the other hand, did not help children to develop their cognitive abilities necessary to operate in ill-defined problem spaces. There are a number of possible explanations for this pattern of effects, including the identification process, the quality and the quantity of the program.

A first explanation could be the fact that the identification method applied for the ET program may have been flawed. In fact, we found that the ET participants were above average in traditional intelligence, most of them with an IQ above 130 for the SPM, but showed a traditional creativity level (TCT–DP) that was average or even below average. This suggests that creative children may have been overlooked (misses), because parents and teachers may be harboring a selection bias that results in considering only children with an above-average school performance. According to Freeman (2005), “teachers often kept a mental image of a gifted pupil who would have exceptionally good logical reasoning abilities, quick comprehension, and intellectual curiosity - in combination with good school grades” (p. 82). To him, highly creative children are generally less agreeable in, and less conforming to, conventional school settings than the ones who are simply highly intelligent. This could be the reason for the former not being selected for giftedness programs. Hany and Heller (1990) found that German teachers did not consider creativity as an indicator of giftedness. They concluded that “teachers want to have the successful and ‘easy to handle’ students in their courses. Critical thinking and having original ideas – signs of creativity – are not ranked highly” (p. 76). Sommer et al. (2008) correlated results obtained in tests of intelligence and creativity with corresponding estimates given by parents and teachers. They observed that parents and teachers could better identify abilities associated with high intelligence than they could detect abilities associated with a high level of creativity. Moreover, it may even be considered possible that parents fabricate information that would enhance their children’s chances of being selected for the program (false alarms).

As a consequence of these identification biases, resulting in both misses and false alarms, many highly intelligent but less creative children were selected for the ET program. The expectation for such a sample would be that a creativity enhancing program would have an even greater effect on the creativity of these children as opposed to children beginning with a high creativity level (Besançon and Lubart, 2008). That there is, however, no effect on creativity in the present study, thus suggests that creative thinking was not challenged sufficiently in the ET program (see below). On the other side, regarding the high IQ level in the present sample, some authors suggested (Sternberg and Kaufman, 2010; Sternberg et al., 2011) that a high level of intelligence may even be an obstacle to the development of creativity. For a person who is accustomed to viewing things in a certain way, it becomes more and more difficult to consider a different perspective (Sternberg and Kaufman, 2010). According to Sternberg et al. (2011) individuals with a high IQ may find it difficult to think creatively because of their pronounced analytical abilities; “those who have very high IQs may be so highly rewarded for their IQ-like (analytical) skills that they fail to develop their creative potential, which may then remain latent” (p. 88). This fact may also play a role in the threshold phenomenon, which expects no correlation between intelligence and creative abilities above an IQ of 120 (Welter et al., 2016).

The identification process applied for the ET program may also have promoted a gender bias. Note, that in the present study, 75% of the children identified as gifted were boys. According to Gagné (1993), boys and girls are perceived differently by their peers and teachers when it comes to their capability in many domains. Boys are often perceived to be advanced in mathematical and technical skills, requiring more analytical and convergent thinking, and girls are more frequently considered competent in language skills and socio-affective abilities. Since the identification process for the present sample presented children with tasks requiring more analytical and convergent thinking, more boys than girls may have been considered for the ET program.

A second explanation for this pattern of results found in the present study might be the quality of the ET curriculum. It is possible that the ET activities were simply not adequate to support creative thinking. Besides offering the children cognitive activities, in which the task is to search for the one and only correct solution, tasks should be provided for which there is no readily available response.

A third explanation for the present pattern of results may be that the frequency of the ET meetings may have been sufficient to improve intelligence but not sufficient to improve creativity. Since the ET program took place only once a week and in the other days of the week children attended their normal classes, we may infer further that: what little cognitive abilities for operating in ill-defined problem spaces that were developed in the ET program, were not sufficiently sustained further in normal classroom situations. Traditional school teaching encourages traditional intelligence, i.e., those abilities that help cognition to operate in well-defined problem spaces. In contrast, abilities that help cognition to operate in ill-defined problem spaces would thus have less opportunity to be applied in normal school tasks.

Research on the development of intelligence revealed that a child’s intelligence is positively affected by school attendance (e.g., Ceci, 1991; Neisser et al., 1996; Ceci and Williams, 1997). Research on creativity is less consistent. It was found that the educational environment either fosters the development of creativity or incites its decline (e.g., Torrance, 1968; Charles and Runco, 2000; Chae, 2003; Lubart and Georgsdottir, 2004; Maker et al., 2008).

Renzulli and Renzulli (2010) argued that programs destined for gifted education has been a fertile area of experimentation, because these programs are not overwhelmed with prescribed curriculum guides or traditional educational methods. According to Sternberg et al. (2011) academic skills are undoubtedly important, but they are only part of what leads to the realization of gifted potential. Adjustments made in the ET curriculum, such as increasing the number of activities that foster creative thinking operating in an ill-defined problem space, could be a way to overcome the fact that the traditional schools promote mainly cognitive abilities related to well-defined problem spaces.

Certainly, another approach that would help children to develop their creative potential would be to change the curriculum of the traditional schools. A curriculum which does not promote creative abilities is a worrying educational reality; the lack of opportunities for children to develop creative thinking is troubling (Renzulli and Renzulli, 2010). Creativity has been the common attribute of individuals who have made notable contributions to technological innovations and social improvements (Torrance, 1984). One reason that proves the importance of the enhancement of creative thinking is that “there are challenges within many facets of society to which an immediate or single correct response cannot be found” (Isaksen and Murdock, 1993, p. 16). Educational institutions, therefore, should provide children the opportunities not only to think creatively and to explore the unknown (Torrance, 1972, 1987; Beghetto, 2010; Smith and Smith, 2010), but also to find and formulate problems for which no readily available answer is at hand (Getzels, 1987; Runco, 1994; Carson and Runco, 1999; Kim, 2011). The observations made about creativity in the educational setting are also valid in relation to intelligence measured in ill-defined problem space situations, since the latter compare better to creativity than to intelligence operating in well-defined problem spaces (Welter et al., 2017).

In sum, results of the present evaluation study show the effectiveness of the ET program only in the improvement of intelligence operating in well-defined problem spaces. The outcome that neither ET-children’s creativity scores (TCT-DP) nor their scores of intelligence operating in ill-defined problem spaces (CRT-R) showed an improvement after 1 year of the enrichment program may indicate that problem space is an important issue when interpreting tests results. According to which of the two types of problem spaces presented, different cognitive abilities would be elicited from the same child.

The present findings might be the consequence of factors which may also interact: the ET identification process, which shows a tendency to choose highly intelligent children with only average creativity, and which promotes more boys than girls; and the quality and quantity of the ET curriculum, which may not have provided enough activities to promote creative thinking and cognitive abilities related to ill-defined problem spaces.

## Author Contributions

All authors listed have made substantial, direct, and intellectual contribution to the work, and approved it for publication. TL was the initiator and supervisor of the study; he participated in data analysis and writing, and is the senior as well as the corresponding author. SJ was the co-advisor of the study and participated in data collection, analysis, and writing. MW conducted the study, ran the analyses, and participated in writing. She is the first author; the study is part of her Ph.D. thesis (Welter, 2014), supervised by TL and SJ.

## Conflict of Interest Statement

The authors declare that the research was conducted in the absence of any commercial or financial relationships that could be construed as a potential conflict of interest.
